# Advances and Challenges in Polymer-Based Scaffolds for Bone Tissue Engineering: A Path Towards Personalized Regenerative Medicine

**DOI:** 10.3390/polym16233303

**Published:** 2024-11-26

**Authors:** Samira Farjaminejad, Rosana Farjaminejad, Melika Hasani, Franklin Garcia-Godoy, Majid Abdouss, Anand Marya, Ari Harsoputranto, Abdolreza Jamilian

**Affiliations:** 1 Department of Health Services Research and Management, School of Health and Psychological Sciences, City, University of London, London WC1E 7HU, UK; samira.farjaminejad@city.ac.uk (S.F.); rosana.farjaminejad@city.ac.uk (R.F.); 2 Department of Biomedical Engineering, Central Tehran Branch, Islamic Azad University, Tehran 1955847781, Iran; hasani.melikaa@gmail.com; 3 Department of Bioscience Research, Bioscience Research Center, College of Dentistry, University of Tennessee Health Science Center, 875 Union Avenue, Memphis, TN 38163, USA; fgarciagodoy@gmail.com; 4 Department of Chemistry, Amirkabir University of Technology (AUT), Tehran 1591634311, Iran; phdabdouss44@aut.ac.ir; 5 Deputy-Dean of Dentistry (Research) & Program, Director of Orthodontics, Faculty of Dentistry, University of Puthisastra, Phnom Penh 55 180, Cambodia; amarya@puthisastra.edu.kh; 6 City of London Dental School, University of Bolton, London BL3 5AB, UK; ari@orthosociety.com; 7 Orthodontic Department, Faculty of Dentistry, University of Puthisastra, Phnom Penh 55 180, Cambodia; 8 Orthodontic Department, Faculty of Dentistry, Tehran Medical Sciences, Islamic Azad University, Tehran 1417935840, Iran

**Keywords:** polymers, regenerative medicine, tissue engineering, bone regeneration, scaffolds

## Abstract

Polymers have become essential in advancing bone tissue engineering, providing adaptable bone healing and regeneration solutions. Their biocompatibility and biodegradability make them ideal candidates for creating scaffolds that mimic the body’s natural extracellular matrix (ECM). However, significant challenges remain, including degradation by-products, insufficient mechanical strength, and suboptimal cellular interactions. This article addresses these challenges by evaluating the performance of polymers like poly(lactic-co-glycolic acid) (PLGA), polycaprolactone (PCL), and polylactic acid (PLA) in scaffold development. It also explores recent innovations, such as intelligent polymers, bioprinting, and the integration of bioactive molecules to enhance scaffold efficacy. We propose that overcoming current limitations requires a combination of novel biomaterials, advanced fabrication techniques, and tailored regulatory strategies. The future potential of polymer-based scaffolds in personalised regenerative medicine is discussed, focusing on their clinical applicability.

## 1. Introduction

Bone tissue engineering aims to repair and regenerate damaged bones through polymer-based scaffolds that mimic the extracellular matrix (ECM). These scaffolds provide structural support and biological cues for essential cellular activities, including attachment, proliferation, and differentiation [1].

By incorporating biocompatible and biodegradable materials, nanoparticles, and growth factors, these scaffolds address the limitations of traditional methods like autografts and allografts by facilitating cell infiltration, nutrient exchange, and bioactive molecule delivery to enhance bone healing [1,2].

Polymers such as PLA and PCL are commonly used because they can be customised for specific mechanical properties, porosity, and bioactivity. This makes them adaptable to complex bone defects and essential for effective tissue regeneration [3,4,5]. Bone consists of cortical bone, a dense outer layer for strength, and trabecular bone, a spongy interior for shock absorption. Cortical bone resists bending and torsion, while trabecular bone provides flexibility in high-stress areas like joints. Bone remodelling involves osteoblasts, which form new bone, and osteoclasts, which resorb old tissue to maintain balance and adapt to mechanical forces [6].

Bone healing follows a complex multi-stage process, beginning with an inflammatory response where blood vessels and soft tissues at the fracture site form a blood clot or hematoma. This is a scaffold for immune cells like neutrophils and macrophages, which clear debris and release signals to recruit stem cells [7]. These stem cells differentiate into chondrocytes and osteoblasts, forming a soft callus and stabilising the fracture. The soft callus later calcifies into a hard callus, providing stability as new bone forms. Over time, osteoclasts and osteoblasts remodel this hard callus into the mature bone, restoring strength and structure, though complete remodelling may take months to years [7].

In bone tissue engineering, scaffolds serve as temporary 3D support for osteoblasts to adhere, proliferate, and differentiate into bone-forming cells. As cells form new bone tissue, the scaffold gradually degrades, allowing it to be replaced seamlessly by the new tissue. This controlled degradation rate, tuned to match tissue growth, ensures structural support without interfering with natural regeneration [8,9].

Modern scaffold designs incorporate bioactive materials, such as bone morphogenetic proteins (BMPs), to stimulate cellular processes essential for healing. This integration enhances the scaffold’s functionality in supporting bone tissue engineering [6,7].

Polymers are integral in scaffold development due to their unique properties—biocompatibility, biodegradability, and mechanical strength. These polymers minimise immune responses, ensuring smooth integration with surrounding tissues, while their surfaces can be modified to promote cell adhesion and proliferation. Their gradual degradation aligns with tissue growth, supporting the healing process until it is no longer needed, eliminating surgical removal [8,9,10,11,12].

Mechanical strength is critical, especially in bone tissue engineering, where scaffolds must withstand physiological loads. Polymers can be engineered to mimic bone’s mechanical properties, providing the necessary flexibility and strength to maintain scaffold integrity throughout healing [3,11].

Additionally, polymers can incorporate bioactive molecules, such as growth factors or drugs, directly within the scaffold to enhance functionality and accelerate regeneration [12,13].

**Figure 1:**
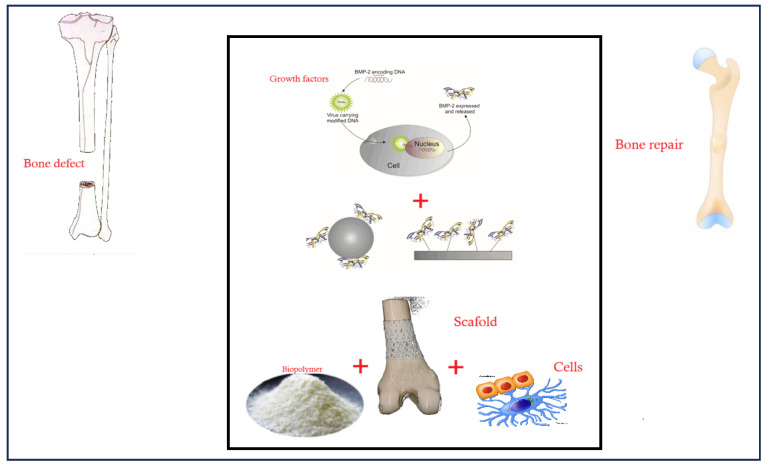
Schematic representation of bone tissue engineering approach for repairing bone defects. The process combines biopolymers, scaffolds, growth factors, and cells to stimulate bone repair and regeneration. The “+” signs in the figure represent the addition of individual components—growth factors, biopolymers, scaffolds, and cells—into the engineered construct.

This article explores the role of polymers in bone tissue engineering, highlighting advancements in scaffold designs, applications, and potential limitations. Bone tissue engineering combines biopolymers, scaffolds, growth factors, and cells to replicate natural healing. For instance, biopolymers provide a biodegradable and biocompatible framework. At the same time, growth factors like BMP-2 promote stem cell differentiation into osteoblasts, optimising the scaffold’s support for cell attachment, proliferation, and new tissue formation. These constructs are essential for restoring damaged bone’s structural and functional integrity, as illustrated in Figure 1.

## 2. Type of Polymers in Bone Tissue Engineering

**Figure 2:**
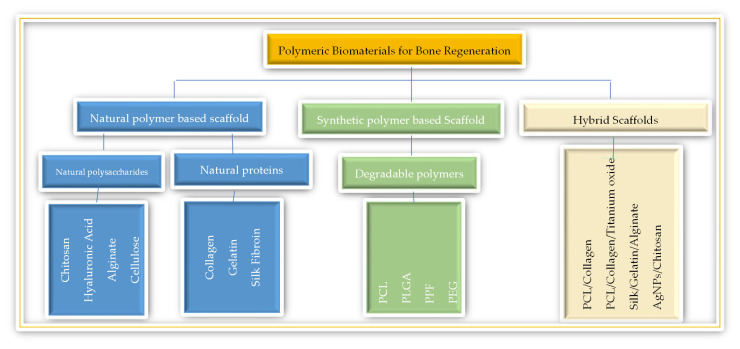
Classification of polymeric biomaterials for bone regeneration.

In bone tissue engineering, polymers are categorised into natural and synthetic types (Figure 2). Natural polymers like collagen, chitosan, and alginate mimic the ECM and are highly biocompatible and biodegradable, promoting cell adhesion and proliferation. However, they often lack the mechanical strength required for load-bearing applications and degrade rapidly [14]. Synthetic polymers, such as PCL, PLA, and PLGA, offer tunable properties, controlled degradation rates, and greater mechanical strength, making them suitable for long-term use. However, they are less bioactive and often need modification or combination with bioactive materials to improve cell attachment and osteoconductive performance [14,15].

### 2.1. Natural Polymers

Due to their similarity to the ECM in bone tissue, natural polymers play a significant role in bone regeneration (Table 1). These polymers, including collagen, chitosan, alginate, and silk fibroin, offer biocompatibility, bioactivity, and biodegradability, making them ideal for scaffolding materials that support bone healing. Collagen, the most abundant protein in the human body, is particularly useful in creating scaffolds that promote cell adhesion and proliferation. It provides structural support while mimicking the native bone environment, aiding natural regeneration. However, natural polymers like collagen often require enhancements, such as cross-linking with other materials, to improve mechanical strength and stability, as they generally lack the rigidity needed for bone regeneration [15].

Chitosan and alginate, both polysaccharide-based polymers, are also widely used for their biodegradability and ability to form hydrogels that facilitate cell migration and nutrient diffusion. Despite their excellent biocompatibility, these natural polymers often have limitations, such as weak mechanical properties, which are addressed by combining them with other materials like calcium phosphate or synthetic polymers to enhance their osteoconductivity and mechanical strength [16].

#### 2.1.1. Collagen-Based Scaffolds for Bone Tissue Engineering

Collagen is a natural polymer widely used in bone tissue engineering due to its biocompatibility and ability to mimic the ECM. It constitutes 90% of the organic components of the bone ECM, making it a critical structural component in bone tissue. Synthetic bone grafts aim to replicate this ECM to support cell attachment, proliferation, and differentiation. However, pure collagen lacks the mechanical strength needed for load-bearing applications. To address this, collagen is often combined with materials like hydroxyapatite (HA) or synthetic polymers such as PCL to enhance mechanical properties and promote mineralisation [16,17].

For example, magnesium-doped hydroxyapatite (MgHA) combined with collagen fibres creates hybrid scaffolds that closely mimic the natural bone ECM, providing a supportive microenvironment for cellular attachment, proliferation, and differentiation. These composites exhibit high porosity, essential for nutrient diffusion and cell migration, while the mineral phase strengthens the scaffold and enhances its bioactivity [18].

The addition of PCL to collagen scaffolds improves their structural integrity while maintaining their biocompatibility. Recent studies have explored the incorporation of hydroxyapatite (HA) into collagen-PCL composites, creating hybrid scaffolds that closely mimic the natural bone ECM. For example, electrospun three-layered scaffolds with an outer PCL layer, a middle PCL-collagen layer, and an inner PCL-collagen-HA layer have shown exceptional promise in guided bone regeneration. These scaffolds not only provide mechanical strength but also promote osteointegration and support osteogenic differentiation. The inclusion of HA enhances the bioactivity of the scaffold, facilitating cell attachment and new bone formation, as evidenced in both in vitro studies and critical-size cranial defect models [19].

**Figure 3:**
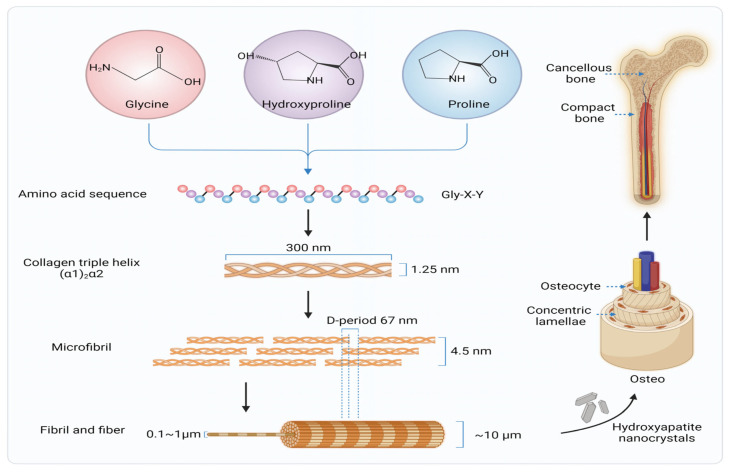
Hierarchical organisation of Type I collagen fibres in human bone [20].

The structure and synthesis of collagen further underscore its role in bone regeneration. Collagen synthesis involves fibroblasts and osteoblasts producing triple helical structures from alpha chains, which self-assemble into microfibrils, fibrils, and fibres with the characteristic 67 nm D-period. These fibrils act as a framework for HA nanocrystal deposition, forming the hierarchical bone matrix. The bone matrix is composed of 65% mineral phase (HA), 35% organic phase (~90% type I collagen, 5% non-collagenous proteins, and 2% lipids), and residual water. This interplay between collagen and HA provides bone with its unique combination of rigidity and toughness (Figure 3). Scaffolds designed for bone regeneration must replicate this natural structure to achieve optimal functionality [20].

Electrospinning allows for creating porous collagen scaffolds that support cell attachment and proliferation, which is critical for bone regeneration. However, the mechanical strength of electrospun collagen remains a challenge, especially in wet environments [16]. Cross-linking methods and the incorporation of synthetic polymers like PCL have improved the durability and functionality of these scaffolds, making them more suitable for clinical applications [16,17].

Despite these advancements, issues such as controlling degradation rates and minimising inflammatory responses remain. Emerging technologies like 3D-printing offer potential solutions for customising scaffold properties [16]. Continued innovation in collagen-based scaffolds holds promise for their wider clinical use in bone regeneration [17].

#### 2.1.2. Chitosan

Chitosan, a naturally derived polymer from chitin, has become increasingly significant in bone tissue engineering due to its excellent biocompatibility, biodegradability, and osteoconductivity. Chitosan is known for promoting cell adhesion and proliferation, making it an ideal candidate for bone regeneration scaffolds [21]. Bavariya et al., 2013, evaluated genipin-crosslinked chitosan nanofibre membranes for bone tissue engineering, finding them comparable to collagen in biocompatibility but with a slower degradation rate, lasting up to 20 weeks. This controlled, extended breakdown makes chitosan particularly suitable for guided bone regeneration, providing sustained structural support for healing significant bone defects [22].

The cationic nature of chitosan allows for electrostatic interactions, which are beneficial for forming cross-linked hydrogels that can act as scaffolds in bone tissue engineering applications. Genipin, a natural crosslinker derived from *Gardenia jasminoides*, has been widely used to enhance the properties of chitosan scaffolds. Genipin-crosslinked chitosan (GEN-chitosan) scaffolds demonstrate superior structural integrity, cytocompatibility, and tunable porosity, which are essential for supporting cell adhesion, proliferation, and differentiation. The crosslinking reaction between genipin and chitosan forms stable covalent bonds, leading to improved resistance to enzymatic degradation and enhanced mechanical properties. This durability makes GEN-chitosan scaffolds particularly effective for long-term applications in bone tissue engineering [23].

The degree of deacetylation (DA) of chitosan plays a crucial role in its solubility, viscosity, and mechanical properties, which can be tailored to meet the specific needs of different tissue engineering applications. Higher DA typically leads to better elasticity, while lower DA results in enhanced mechanical strength [21].

GEN-chitosan scaffolds further benefit from tailored porosity and stiffness, providing an optimised environment for cell infiltration, extracellular matrix deposition, and osteogenic differentiation. These properties make them particularly effective for applications such as guided bone regeneration and load-bearing scaffolds [23].

Recent advancements have further enhanced chitosan’s versatility through the incorporation of nanomaterials. For example, mesoporous silica nanoparticles (MSNs) have been incorporated into chitosan-alginate composite scaffolds, resulting in improved mechanical strength, swelling behaviour, and osteogenic potential while maintaining high biocompatibility. The porous structure created using freeze-drying methods supports cell attachment, proliferation, and mineral deposition, making these scaffolds promising for craniofacial and orthopaedic applications [24].

**Figure 4:**
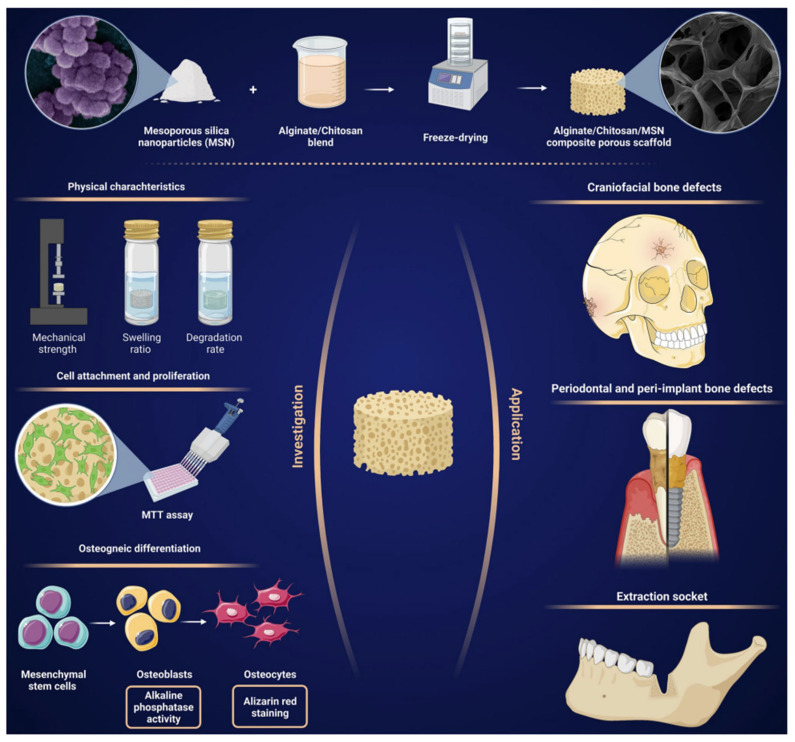
Schematic representation of the synthesis, characterisation, and application of alginate/chitosan/mesoporous silica nanoparticle (MSN) composite scaffolds for bone tissue engineering. The scaffolds are fabricated using a freeze-drying process, resulting in a porous structure with enhanced mechanical strength, swelling capacity, and controlled degradation. Biological evaluations include cell attachment and proliferation assays (MTT assay) and osteogenic differentiation assessments using alkaline phosphatase activity and Alizarin red staining. The scaffolds show potential for applications in craniofacial bone defects, periodontal and peri-implant bone defects, and tooth extraction sockets. Adopted from [24].

Figure 4 illustrates the fabrication and application of MSN-incorporated chitosan-alginate scaffolds. This schematic highlights the porous scaffold structure, which supports critical features like mechanical strength, nutrient transport, and osteogenic activity, aligning with the needs of bone tissue engineering. Such innovations underscore the potential of chitosan-based scaffolds in addressing challenges associated with bone regeneration [24].

Moreover, chitosan’s ability to form physical and chemical hydrogels enhances its versatility in scaffold design, allowing it to be easily combined with other materials like hydroxyapatite, which improves its osteoconductive properties. Recent advancements have been explored by combining chitosan with nanomaterials, such as graphene oxide or calcium phosphate, to enhance its mechanical properties and promote bone regeneration. Chitosan-based hydrogels have been shown to support cell infiltration, growth, and osteogenic differentiation, making them a promising material for bone regeneration [21].

#### 2.1.3. Gelatin and Alginate

Gelatin and alginate are two critical natural polymers extensively used in bone tissue engineering due to their favourable biocompatibility, biodegradability, and ability to form hydrogels. Gelatin, a collagen derivative, contains the Arg-Gly-Asp (RGD) sequence, which promotes cell adhesion, proliferation, and differentiation. This makes it an effective scaffold material for bone regeneration. However, gelatin alone lacks the mechanical strength required for load-bearing applications, so it is often combined with other materials like alginate for improved stability and structural integrity [25].

Alginate, derived from brown algae, is widely used in bone tissue engineering due to its biocompatibility and accessible cross-linking properties. However, alginate lacks inherent cell adhesion motifs and is often chemically modified or combined with gelatin to enhance its bioactivity. Together, gelatin and alginate form hydrogels that support cell encapsulation and tissue growth. Their combination, especially in 3D-bioprinting applications, provides scaffolds with improved mechanical strength and bioactivity, essential for successful bone regeneration [25].

#### 2.1.4. Hyaluronic Acid (HA)

HA has emerged as a valuable natural polymer in bone tissue engineering due to its biocompatibility, biodegradability, and ability to mimic the ECM. HA plays a crucial role in wound healing and tissue hydration, making it an excellent candidate for supporting bone regeneration [26,27]. In bone repair, HA can serve as a scaffold or carrier for bioactive molecules, such as growth factors and osteogenic cells, enhancing osteogenesis and mineralisation [28,29]. HA’s effectiveness is primarily attributed to its ability to interact with cell surface receptors, such as CD44, which promotes cell adhesion, proliferation, and migration. This interaction is essential for recruiting mesenchymal stem cells and osteoprogenitor cells to the site of injury, aiding in the formation of new bone tissue [28,29]. Moreover, HA can be chemically modified or combined with other biomaterials, such as collagen and ceramics, to form hydrogels or composite scaffolds that improve their mechanical properties and extend their degradation time [22,23].

Despite its potential, HA alone does not significantly enhance bone regeneration. However, when combined with other materials or bioactive agents, such as bone morphogenetic proteins (BMP-2), HA-based scaffolds can significantly improve bone healing outcomes by promoting osteogenic differentiation and improving vascularisation [26]. This makes HA a promising component in developing advanced scaffolds for bone tissue engineering, especially in cases of large or complex bone defects.

#### 2.1.5. Silk Fibroin in Bone Tissue Engineering

Silk fibroin, a protein derived from the cocoons of the *Bombyx mori* silkworm, has garnered significant attention as a promising biomaterial for bone tissue engineering. Its unique combination of biocompatibility, mechanical strength, tunable biodegradability, and ability to support osteogenesis positions it as a favourable scaffold material for bone repair and regeneration [30,31].

One of the standout features of silk fibroin is its mechanical properties, which can be tailored to mimic the ECM of bone tissue. It boasts high tensile strength and elasticity, providing essential structural support during healing [30]. Furthermore, the degradation of silk fibroin is controllable, meaning it can be designed to break down and be replaced by regenerating tissue gradually. This characteristic is crucial for bone scaffolds, as the scaffold degradation rate ideally aligns with the rate of new bone formation [25,27].

Silk fibroin’s versatility extends to its processing methods. It can be fabricated into various scaffold forms such as gels, sponges, and composite materials (silk). In bone tissue engineering, silk fibroin is often combined with inorganic materials like hydroxyapatite (HAP) to create hybrid scaffolds that mimic the composite nature of bone, consisting of organic and inorganic components [31,32]. These silk fibroin-hydroxyapatite composites have enhanced osteoconductivity and bioactivity, promoting cell attachment, proliferation, and differentiation. Studies have demonstrated that silk fibroin facilitates the nucleation and growth of HAP crystals, improving the biomineralisation process essential for bone regeneration [27].

Additionally, silk fibroin’s surface can be chemically modified to enhance its interaction with cells and growth factors. This adaptability makes it suitable for personalised regenerative medicine, where scaffolds need to cater to the specific requirements of individual patients [30].

Despite these advantages, scaling up production and ensuring consistent quality remain challenges. Furthermore, optimising the balance between mechanical strength, biodegradation, and bioactivity in clinical applications requires further research [25,27]. However, as research advances, silk fibroin holds great potential as a critical material in the future of bone tissue engineering.

#### 2.1.6. Cellulose in Bone Tissue Engineering

Cellulose, a natural polysaccharide abundantly found in plants, algae, and some bacteria, has gained recognition in bone tissue engineering due to its favourable properties such as biocompatibility, biodegradability, and non-toxicity. Its structure, composed of glucose units linked by β-1,4-glycosidic bonds, offers mechanical strength and stability, making it an excellent candidate for scaffolds aimed at bone regeneration [33,34]. In bone tissue engineering, cellulose-based scaffolds have demonstrated potential in supporting cell attachment, proliferation, and differentiation. By mimicking the natural ECM, these scaffolds provide an optimal environment for tissue regeneration. Additionally, cellulose-based materials can be modified to enhance their mechanical properties and biodegradability, allowing for more effective integration into the body and supporting long-term bone healing [34,35].

**Table 1:** Comparative analysis of common natural polymers in bone tissue engineering.

Polymer	Key Benefits	Limitations	Ref
Collagen	High biocompatibility, mimicking ECM - Promotes cell adhesion and proliferation - Supports mineralisation	- Lacks mechanical strength for load-bearing applications - Requires crosslinking for improved stability	[16,17]
Chitosan	Encourages cell attachment and osteogenic differentiation - Forms hydrogels suitable for scaffold applications	- High production cost - Variability in quality - Environmental impact from production	[21]
Alginate	- Biodegradable and compatible with bone cells - Cost-effective and easy to modify - Supports 3D-printing applications	Limited mechanical durability - Rapid degradation can lead to insufficient support - No inherent bioactivity	[25,36]
Hyaluronic Acid	- Bioactive, mimicking natural bone ECM - Biodegradable, promoting safe integration	- Weak mechanical properties - Fast degradation rate, limiting prolonged support	[26,27,37]
Silk Fibroin	- Strong mechanical properties and bioactivity -Chemically modifiable for tailored applications	-Environmental sustainability concerns in production - Scaling up for clinical applications remains challenging	[30,31]
Cellulose	- Excellent porosity for cell attachment - Biocompatible and promotes cell proliferation - Supports scaffold integration	- Poor water solubility limits applications - Not naturally degradable in the human body	[34,35].

Various forms of cellulose, such as nanocrystalline cellulose (NCC) and microcrystalline cellulose (MCC), have been explored for their unique structural and mechanical advantages. These nano- and micro-sized particles improve scaffold rigidity and enhance mineralisation, which is crucial for bone formation. Furthermore, cellulose derivatives, including carboxymethyl cellulose (CMC) and bacterial cellulose (BC), have been studied for their ability to form hydrogels and composites that combine the mechanical strength of synthetic polymers with the bioactivity of natural materials [35].

However, despite these advantages, pure cellulose still poses challenges, particularly in its slow degradation rate and limited osseointegration. To address these limitations, cellulose is often combined with other materials like hydroxyapatite, chitosan, or synthetic polymers to create composite scaffolds. These combinations improve the scaffold’s mechanical and biological properties, promoting better bone regeneration and integration [34].

### 2.2. Synthetic Polymers

Synthetic polymers are widely used in bone tissue engineering due to their tunable properties, controlled degradation rates, and structural versatility. They can be designed with specific mechanical and chemical characteristics to meet the needs of bone regeneration. Poly(ε-caprolactone) (PCL), polylactide (PLA), and poly(lactic-co-glycolic acid) (PLGA) are among the most common synthetic polymers used in scaffold development.

PCL is valued for its slow degradation rate, making it suitable for long-term scaffolds in bone regeneration. However, its hydrophobic nature, which affects cell adhesion, is often modified or combined with bioactive materials such as hydroxyapatite to enhance its osteoconductivity [14]. PLA, on the other hand, is biocompatible and thermally stable, making it ideal for use in 3D-printing scaffolds. It can be combined with hydroxyapatite to improve mechanical properties and promote bone growth [14].

PLGA is another highly versatile synthetic polymer offering controlled degradation rates by varying the lactic to glycolic acid ratio. It is often combined with ceramics like hydroxyapatite to enhance osteoconductivity and improve cell attachment. PLGA’s flexibility in scaffold design makes it a widely used material in bone tissue regeneration [14].

#### 2.2.1. Polylactic Acid (PLA)

PLA is one of the most widely used synthetic polymers in bone tissue engineering due to its biocompatibility, biodegradability, and mechanical strength. PLA is biodegradable from renewable resources, breaking down naturally into non-toxic byproducts such as lactic acid, metabolised by the body. This makes PLA an excellent candidate for temporary scaffolds used in bone regeneration [38].

PLA-based scaffolds offer customisable mechanical properties through molecular weight and crystallinity, allowing them to be tailored for specific applications. Poly(L-lactic acid) (PLLA), a more crystalline form of PLA, is often used for load-bearing applications due to its superior mechanical properties, such as higher strength and rigidity. However, one challenge with PLA is its relatively slow degradation rate, which can sometimes lead to inflammation if degradation byproducts accumulate. To mitigate this, PLA is often blended with other polymers like poly (glycolic acid) (PGA) to create copolymers with faster degradation rates [38,39].

PLA’s processability, primarily through 3D-printing, allows for creating scaffolds with precise structures, including controlled porosity and geometry. This enables the scaffolds to support cell infiltration, proliferation, and differentiation, promoting effective bone healing. Furthermore, PLA scaffolds can be enhanced with bioactive molecules or combined with osteoconductive materials like hydroxyapatite (HA) to improve their functionality in bone tissue engineering [38,40].

Overall, PLA’s biocompatibility, customisability, and adaptability to advanced fabrication techniques make it a valuable material for scaffolds in bone tissue regeneration despite some challenges related to its degradation rate [38,40].

#### 2.2.2. Polycaprolactone (PCL)

PCL is a widely used synthetic polymer in bone tissue engineering due to its biocompatibility, biodegradability, and mechanical versatility. PCL is a semi-crystalline polyester approved by the FDA, making it a reliable material for long-term scaffolds in load-bearing bone regeneration applications. One of the primary advantages of PCL is its slow degradation rate, which allows the scaffold to maintain its structural integrity for extended periods, supporting bone healing over time [41,42].

However, PCL’s hydrophobicity can limit cell adhesion and proliferation. To overcome this, PCL is often combined with bioactive materials such as hydroxyapatite (HA) or ceramic micro-powder. These combinations enhance the scaffold’s osteoconductivity and improve its ability to support bone cell growth [42,43]. Studies have shown that PCL/HA composites can significantly improve cell attachment, proliferation, and osteogenic differentiation, making them more effective for bone tissue regeneration [43].

Additionally, PCL is commonly used in 3D-printing to create custom scaffolds tailored to specific bone defects. The precise control over pore size and scaffold architecture in 3D-printed PCL scaffolds enhances their mechanical properties and ensures adequate cellular infiltration and vascularisation, which are essential for bone regeneration [41].

#### 2.2.3. Poly(Lactic-Co-Glycolic Acid) (PLGA)

PLGA is a widely used synthetic polymer in bone tissue engineering due to its biocompatibility, biodegradability, and versatile properties. PLGA is a copolymer made of lactic acid and glycolic acid, and its degradation rate can be controlled by adjusting the ratio of these two monomers. PLGA has found broad applications in bone regeneration, particularly in developing scaffolds, microspheres, and drug-delivery systems [44,45].

One of the primary reasons for PLGA’s popularity is its tunable degradation profile, which allows it to be used for both short- and long-term bone regeneration. The degradation occurs through hydrolysis, and the ratio of lactic to glycolic acid influences the rate. For instance, a 50:50 ratio degrades more rapidly than other compositions, providing flexibility in scaffold design based on the specific needs of the bone defect [44,46].

PLGA is often used with bioactive materials, such as hydroxyapatite (HA), to improve osteoconductivity. HA-PLGA composites have been shown to enhance bone regeneration by supporting better cell attachment, proliferation, and differentiation. Additionally, PLGA is frequently utilised in drug delivery systems. Its ability to encapsulate growth factors, like bone morphogenetic protein-2 (BMP-2), and release them in a controlled manner significantly enhances bone healing [44,46].

Furthermore, PLGA can be processed into various forms, such as microspheres, nanoparticles, and 3D-printed scaffolds, making it a versatile material for bone defect filling and drug delivery. Combined with its FDA approval and wide availability, these properties make PLGA one of the most widely researched polymers for bone tissue engineering [37,45].

#### 2.2.4. Polyethylene Glycol (PEG)

PEG is widely utilised in bone tissue engineering due to its biocompatibility, low immunogenicity, and ability to enhance other materials’ properties when combined. PEG is often combined with other polymers, such as poly(lactic acid) (PLA), to improve their hydrophilicity, thereby enhancing cell adhesion, proliferation, and overall tissue integration [47,48].

One of the critical advantages of PEG in bone tissue engineering is its ability to act as a hydrophilic segment in amphiphilic block copolymers. These copolymers improve the scaffold’s wettability, facilitating better interaction with cells and promoting tissue growth [49]. PEG-based copolymers can also form hydrogels with tunable swelling properties, which aid in nutrient transport and cell migration and are critical for bone regeneration [49].

However, unmodified PEG is non-degradable and lacks bioactivity, which limits its use in isolation for bone scaffolds. To overcome these limitations, PEG is often combined with degradable polymers, such as poly(lactic-co-glycolic acid) (PLGA) or polycaprolactone (PCL), to create bioactive scaffolds that degrade over time, supporting bone regeneration while maintaining structural integrity during the healing process [49]. Additionally, PEG’s ability to reduce protein adsorption and prevent non-specific cell adhesion makes it an ideal candidate for coating scaffolds to control cell interactions [47].

PEG’s combination of hydrophilicity, biocompatibility, and flexibility in modification makes it a valuable polymer in designing scaffolds for bone tissue engineering [48].

#### 2.2.5. Poly(Propylene Fumarate) (PPF)

PPF is a synthetic polymer extensively studied for its potential applications in bone tissue engineering due to its biodegradability, biocompatibility, and cross-linked ability, providing robust mechanical properties. PPF degrades through the hydrolysis of its ester bonds, producing biocompatible byproducts such as fumaric acid and propylene glycol, which are quickly metabolised by the body [50,51].

PPF’s ability to be tailored with bioactive materials enhances its use in bone regeneration scaffolds. For instance, PPF is often combined with bioactive ceramics such as bioactive glass (BG), improving mechanical strength and osteoconductivity. Studies have shown that PPF/BG composite scaffolds promote the formation of hydroxycarbonate apatite layers in simulated body fluid (SBF), which is crucial for supporting osteoblast adhesion and bone tissue growth [52].

Moreover, the mechanical properties of PPF can be customised through crosslinking, allowing it to be used in load-bearing bone repair applications. Microsphere-based PPF scaffolds have gained attention for their ability to deliver bioactive molecules and support cell proliferation and differentiation. These scaffolds can maintain their structure during the bone healing process while gradually degrading [51].

Overall, PPF’s degradation rate, crosslinking potential, and ability to form bioactive composite scaffolds make it a promising material for bone tissue engineering applications [52].

#### 2.2.6. Poly(Glycolic Acid) (PGA)

PGA is a synthetic biodegradable polymer that has garnered significant attention in bone tissue engineering due to its biocompatibility, high degradation rate, and mechanical strength. PGA is one of the earliest FDA-approved polymers used in biomedical applications, particularly in degradable sutures, which underlines its safety and effectiveness in human applications [53,54].

PGA has a fast degradation rate due to its hydrolytic breakdown in aqueous environments. It is suitable for short-term scaffolds where rapid degradation is necessary to match the pace of new tissue formation. The degradation products of PGA, such as glycolic acid, are non-toxic and can be metabolised by the body, which enhances its safety for clinical use [53,55].

In bone tissue engineering, PGA is frequently used with other materials, such as collagen, to improve its biological properties. For example, collagen-PGA composites provide a scaffold that combines the strength of PGA with the biological benefits of collagen, enhancing cell attachment, proliferation, and osteogenic differentiation [54,55]. Additionally, these composites address the issue of the rapid degradation of PGA by adding collagen’s slower degradation rate, ensuring scaffold integrity during the critical early stages of bone healing [55].

PGA is also incorporated into 3D-printed scaffolds due to its excellent processability. This allows for precise control over scaffold architecture, pore size, and porosity. This is essential for promoting vascularisation and ensuring that cells can infiltrate and populate the scaffold, leading to better tissue integration and bone regeneration [53,54].

Overall, PGA’s rapid degradation, non-toxicity, and ability to form composite materials with improved biological properties make it a valuable polymer in bone tissue engineering. However, due to its fast degradation, it is often combined with other polymers or materials to optimise its performance for longer-term applications [53].

Synthetic polymers such as PLA, PCL, and PLGA offer advantages in biodegradability and mechanical strength but face limitations like hydrophobicity or slow degradation rates. Conversely, natural polymers like collagen, chitosan, and hyaluronic acid excel in biocompatibility but often require enhancement for sufficient mechanical strength (Table 2).

**Table 2:** Comparative analysis of polymer types.

Polymer	Biocompatibility	Degradation Rate	Mechanical Strength	Clinical Applications	
Natural Polymers					
Collagen	Very high	Fast	Low	Non-load-bearing, cell adhesion	[16,17]
Chitosan	High	Moderate	Moderate	Hydrogels, cell attachment support	[21]
Gelatin	High	Fast	Low	Hydrogels, cell adhesion	[25]
Alginate	High	Moderate	Low	Hydrogels, 3D-bioprinting	[25]
Hyaluronic Acid	Very high	Fast	Low	Scaffold for bioactive molecule delivery	[26,27]
Silk Fibroin	High	Moderate	High	Load-bearing applications, ECM mimicry	[30,31]
Cellulose	High	Slow	Moderate	Cell adhesion, scaffold support	[33,34]
Synthetic Polymers					
PEG	High	Non-degradable	Low	Scaffold coatings hydrogels for cell migration	[47,48]
PPF	High	Moderate	High	Load-bearing scaffolds, composite scaffold support	[50,51]
PGA	Moderate	Fast	High	Rapidly degrading scaffold, early-stage support	[53]
PLAY	High	Slow	Moderate	Load-bearing applications	[38,39]
PCL	Moderate	Very slow	High	Long-term scaffold support	[41,42]
PLGA	Moderate (tunable)	Fast (adjustable)	Moderate	Short-term scaffolds, drug delivery	[44,45]

## 3. Role of Polymers in Cellular Interactions

Polymers in bone scaffolds provide physical support and influence cellular interactions crucial for tissue growth. For instance, polymers can be engineered to enhance cell adhesion by introducing surface functionalities that interact with cell receptors like integrins. Additionally, incorporating growth factors into these polymers can stimulate pathways like the BMP/Smad signalling cascade, promoting osteogenic differentiation. Polymers are often combined with bioactive ceramics like hydroxyapatite for enhanced osteoconductivity, which mimics bone mineral composition and encourages cellular responses aligned with bone formation [53].

## 4. Applications and Recent Advances in Polymer-Based Bone Tissue Engineering

### 4.1. 3D-Printing with Polymers for Custom Bone Scaffolds in Tissue Engineering

The application of 3D-printing with polymers in bone tissue engineering has transformed the fabrication of scaffolds, providing highly customisable and patient-specific solutions. By employing additive manufacturing (AM), polymers such as PLA, PCL, and PEG are commonly used to create scaffolds that support bone regeneration [56,57]. This technique allows for precise control over scaffold architecture, including pore size, shape, and mechanical properties. These are essential for mimicking the natural ECM and enhancing cell attachment and tissue growth.

The use of 3D-printing enables the creation of scaffolds with interconnected porosity, promoting better nutrient flow, waste removal, and cell infiltration—critical factors for effective bone healing [56]. Polymers like PLA and PCL are widely used to develop scaffolds with sufficient mechanical strength while supporting bone tissue regeneration. Additionally, hydrogels based on PEG can be combined with 3D-printing to create bioactive scaffolds that can incorporate cells and growth factors [57].

Recent advancements have also enabled the integration of growth factors and drugs directly into the printed scaffolds, allowing for the controlled release of bioactive molecules such as bone morphogenetic proteins (BMPs) to accelerate bone healing [58]. Combining polymers with bioactive ceramics, like hydroxyapatite (HA), further enhances scaffold osteoconductivity, promoting bone tissue formation and regeneration [59].

Enhancing the vascularisation of polymer scaffolds to ensure adequate nutrient supply in larger bone defects involves several strategies. Incorporating angiogenic growth factors like VEGF and bFGF, co-culturing with vascular cells, and using bioactive materials such as hydroxyapatite can significantly promote blood vessel formation. Additionally, 3D-bioprinting techniques to create pre-vascularised networks, gene therapy to introduce angiogenic factors, and nanotechnology to modify scaffold surfaces can further enhance vascularisation. These combined approaches ensure better nutrient delivery and improve the success of bone regeneration [60].

Achieving uniform cell distribution within 3D-printed polymer scaffolds presents several challenges. Static cell-seeding processes often result in heterogeneous cell distribution due to inefficient methods, while restrictive pore sizes and scaffold geometries hinder cell infiltration and movement. To overcome these limitations, dynamic seeding techniques, such as perfusion bioreactors, can be employed to enhance cell distribution by continuously circulating the cell suspension through the scaffold. Optimising scaffold design by adjusting pore size, shape, and interconnectivity facilitates better cell migration and distribution, ensuring a more even distribution of cells within the scaffold [61,62].

Additionally, applying bioactive coatings to the scaffold can enhance cell adhesion and proliferation, leading to more uniform cell distribution. Incorporating cells directly into the bioink during the 3D-printing process ensures a more uniform initial cell distribution. Alternatively, embedding cells within hydrogels before integrating them with the scaffold provides a supportive environment for cells and can be designed to degrade at controlled rates, releasing cells gradually into the scaffold. These approaches significantly improve the uniformity of cell distribution, enhancing the effectiveness of scaffolds in tissue engineering applications [61,62]

The use of 3D-printing in bone tissue engineering facilitates the creation of scaffolds that match the patient’s bone geometry, providing the necessary mechanical support while fostering an environment conducive to bone regeneration [56,58].

### 4.2. Nanotechnology-Enhanced Polymer Scaffolds for Bone Healing and Drug Delivery

Nanotechnology in bone tissue engineering involves the incorporation of nanoparticles (NPs) into polymer matrices to enhance bone healing by improving cell attachment, providing drug delivery, and promoting better overall scaffold performance. Various types of NPs, including ceramics, metals, and polymers, are used to create nanocomposite scaffolds, offering unique properties that mimic the natural ECM of bone tissue [2,63].

Nanoparticles can be incorporated into scaffolds to improve mechanical properties, making them more suitable for load-bearing applications in bone regeneration. Nanohydroxyapatite (nano-HA), for example, enhances scaffolds’ osteoconductivity and osteogenic potential due to its biocompatibility and ability to mimic the mineral phase of natural bone [2]. This allows for better cell attachment and proliferation, essential for bone healing.

In addition to enhancing mechanical properties, NPs can serve as drug delivery systems. Nanoparticles like PLGA and PEG can encapsulate bioactive molecules such as bone morphogenetic proteins (BMPs) or other growth factors, allowing for controlled and sustained release over time, which helps to accelerate bone regeneration [2,64,65]. This method ensures that the therapeutic agents are delivered directly to the bone defect site, improving efficacy while reducing side-effects typically associated with systemic delivery [64].

Furthermore, metallic nanoparticles, such as gold and silver NPs, exhibit antimicrobial properties, which help prevent infections at the implant site [2,64]. Carbon-based nanoparticles, such as graphene and carbon nanotubes, enhance scaffolds’ mechanical strength and biological performance, promoting faster bone repair [2].

Through these advancements, nanotechnology offers versatile and highly effective strategies to improve scaffold performance, support bone tissue regeneration, and provide targeted drug delivery. Integrating nanoparticles into polymer matrices is rapidly becoming a crucial approach in developing more effective bone tissue engineering solutions [2,63,64].

### 4.3. Growth Factors and Drug Delivery via Polymer Scaffolds in Bone Tissue Engineering

Growth factors and drug delivery in bone tissue engineering enhance bone regeneration. Polymer-based scaffolds are widely used as drug delivery systems to control the release of bioactive molecules such as bone morphogenetic proteins (BMPs), growth factors, and other therapeutic agents. These scaffolds provide mechanical support for bone growth and deliver these molecules directly to the site of injury, promoting healing and osteogenesis [66,67].

One of the most researched growth factors in bone tissue engineering is BMP-2. This powerful osteoinductive protein stimulates the differentiation of mesenchymal stem cells into osteoblasts, which are responsible for bone formation. Incorporating BMP-2 into polymer-based scaffolds, such as PLGA, allows for its controlled release, ensuring that the protein is delivered over time at the required concentration for bone regeneration [67]. This controlled release is essential because delivering BMP-2 in excessive amounts can lead to complications such as ectopic bone formation or other adverse effects [67].

Polymers like PLGA and PEG are commonly used for their biodegradability and biocompatibility, which ensure that the scaffold degrades after fulfilling its function, leaving behind no harmful residues [67]. These polymers can be engineered to release growth factors in response to environmental stimuli, such as changes in pH or temperature, making them highly effective in targeted therapies [67]. Drug-loaded polymer scaffolds have been used to treat bone disorders. They deliver anti-inflammatory or antimicrobial agents to the defect site, further enhancing the healing process [66].

Recent advances in composite scaffolds combine bioceramics, like hydroxyapatite (HA), with biodegradable polymers to create osteoconductive structures that mimic natural bone while delivering drugs or growth factors [67]. This combination enhances the scaffold’s mechanical strength and its ability to promote bone healing through the sustained release of bioactive molecules [67].

These polymer-based systems represent a significant advancement in bone tissue engineering. They offer the dual function of mechanical support and bioactive molecule delivery, both of which are essential for efficient and effective bone regeneration [66,67].

### 4.4. Hybrid Scaffolds: Combining Natural and Synthetic Polymers for Enhanced Bone Regeneration

Hybrid scaffolds in bone tissue engineering combine natural and synthetic polymers to balance mechanical strength and biological function, creating scaffolds that promote structural stability and tissue regeneration. These scaffolds integrate the mechanical properties of synthetic materials, such as PCL, with the bioactivity of natural polymers like collagen (Col) and alginate, providing a synergistic effect for bone regeneration [68].

Combining PCL and nano-hydroxyapatite (nHA) in hybrid scaffolds offers enhanced osteoconductivity and mechanical stability, which are critical for supporting bone growth in load-bearing applications. On the biological side, integrating natural polymers like alginate and collagen promotes cell attachment, proliferation, and osteogenic differentiation, supporting the healing process. These bioactive components mimic the ECM, offering a conducive environment for stem cells, such as human dental pulp stem cells (hDPSCs), to differentiate into bone cells [68].

Research shows that hybrid scaffolds, such as those made from PCL/nHA combined with a bioactive hydrogel of alginate, collagen, and nHA, improve both cell viability and mineral deposition during the bone regeneration process. By integrating bioactive hydrogels within the scaffold’s porous structure, hybrid scaffolds also enhance vascularisation and tissue integration, overcoming one of the main challenges in bone tissue engineering [68].

Recent studies emphasise the potential of these hybrid scaffolds to treat critical-size bone defects, offering a promising foundation for clinical applications in bone repair and regeneration [68].

### 4.5. Applications of Polymers in Dental and Bone Tissue Engineering

**Figure 5:**
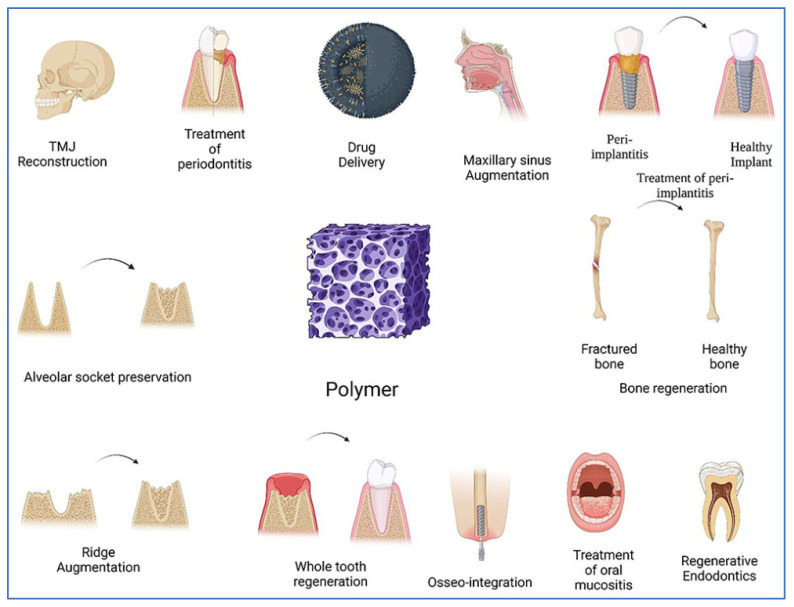
Applications of polymer-based materials in dental and bone tissue engineering. Polymers serve diverse roles, including TMJ reconstruction, periodontitis treatment, drug delivery, maxillary sinus augmentation, alveolar socket preservation, and ridge augmentation. They support bone regeneration, osseointegration, and whole-tooth regeneration and provide scaffolds for regenerative endodontics and treating peri-implantitis and oral mucositis. These versatile materials offer promising solutions for complex dental and orthopaedic challenges [69].

Polymeric biomaterials are widely used in oral, dental, and maxillofacial reconstruction (Figure 5). Photopolymerisable and biodegradable polymers have proven valuable for various dental applications, providing versatile options for reconstruction and tissue regeneration. The photo-triggered polymerisation technique allows for the development of injectable photopolymerisable biomaterials, which are beneficial for dentistry and other regenerative fields. This approach creates a supportive microenvironment conducive to cell proliferation and tissue formation, facilitating the manufacture of scaffolds and the assembly of complex systems. Biomaterials developed through this technique range from fully synthetic polymers, like polyethene glycol, to naturally derived ones, such as hyaluronic acid. Photopolymerisable biomaterials enable the creation of injectable, multifunctional scaffolds that can deliver growth factors, cells, drugs, and other therapeutic agents to enhance tissue regeneration and repair [69].

## 5. Challenges and Limitations

Polymer-based scaffolds face significant challenges in bone tissue engineering, limiting their clinical applicability. These challenges include biodegradation, mechanical strength, cellular interactions, and scalability [1,67,70]. The degradation characteristics of polymer scaffolds are multifaceted and depend on factors such as the type of polymer used, the degradation rate, and the biological environment.

The primary challenges with polymer-based scaffolds include degradation byproducts, mechanical strength limitations, and insufficient bioactivity. Synthetic polymers like PLA and PGA produce acidic degradation byproducts, which, if not managed carefully, can lead to localised inflammation and inhibit tissue growth [1,53]. For example, scaffolds made from fast-degrading polymers such as PLGA may initially support bone formation but can result in reduced bone volume over time due to premature degradation. On the other hand, slowly degrading polymers like PCL can maintain their structural integrity for extended periods, providing sustained support for bone growth and remodelling [71]. However, these materials must ensure their degradation products are biocompatible and non-toxic to avoid adverse effects on surrounding tissues. Accumulation of acidic degradation products, for instance, can lead to local pH changes, negatively impacting cell viability and bone health [72]

Additionally, the immune response to scaffold degradation products is a critical factor. Inflammatory reactions triggered by the presence of degradation by-products can impair tissue growth and bone healing. Designing scaffolds with controlled degradation rates and biocompatible materials is essential to minimize these risks. For example, combining polymers with bioactive ceramics like nano-hydroxyapatite (n-HA) improves scaffold bioactivity and reduces the risks associated with polymer degradation by-products. These composite materials can also buffer acidic by-products, further enhancing scaffold compatibility and functionality [67,70].

Achieving optimal porosity while maintaining the scaffold’s structural integrity is another crucial factor for effective bone tissue regeneration. High porosity allows for better cell infiltration and nutrient diffusion but often compromises the mechanical strength needed for load-bearing applications [58]. Balancing these factors requires further research to develop scaffolds that can provide adequate mechanical support while promoting cellular activities essential for bone growth [53].

Another critical limitation is ensuring the scaffold’s degradation rate aligns with the pace of new bone formation. If the scaffold degrades too quickly, it may fail to provide sufficient support for new tissue; conversely, a slower degradation rate can interfere with natural tissue remodelling. Continued advancements in scaffold materials and design, including bioactive coatings and immunomodulatory molecules, are essential to overcome these hurdles. Embedding growth factors or anti-inflammatory agents within scaffolds can further support tissue integration and mitigate adverse immune responses [53,67,70].

Overall, the long-term effects of polymer scaffold degradation products must be carefully managed to ensure stability, functionality, and compatibility with the biological environment. These considerations are critical for optimising scaffold performance and improving clinical outcomes in bone tissue engineering.

## 6. Prospects

The future of bone tissue engineering is filled with promising advancements, particularly in intelligent polymers, bioprinting, gene therapy integration, and personalised medicine.

Smart polymers represent a significant step forward, as they can respond to environmental stimuli such as pH, temperature, or mechanical stress. These materials hold great potential for controlled release systems, where growth factors, drugs, or bioactive molecules are delivered precisely when and where needed. Recent developments have introduced shape-memory polymers, which can alter their structure in response to temperature changes, making them especially valuable in minimally invasive surgeries. Once implanted, these polymers adapt to their environment, providing mechanical support and promoting tissue integration [73]. As these materials are further refined, they are expected to more accurately mimic natural tissue responses and evolve dynamically throughout the healing process, creating dynamic scaffolds that improve the effectiveness of bone regeneration by better-supporting tissue integration and mechanical stability [2].

Bioprinting has also seen significant advancements, enabling the precise deposition of cells, biomaterials, and growth factors within scaffolds. This technology allows for the production of complex, patient-specific scaffolds that replicate the architecture of natural bone tissue. Through inkjet extrusion and laser-assisted bioprinting techniques, scaffolds are printed layer-by-layer, incorporating living cells and bioactive molecules such as bone morphogenetic proteins (BMPs). These bio-inks promote controlled cell placement and the gradual release of growth factors, fostering better bone growth and healing [70]. Additionally, integrating vascular networks into these scaffolds ensures effective nutrient delivery and waste removal during the healing process, which is crucial for successful tissue regeneration [70]. The ability to control scaffold properties such as pore size, shape, and interconnectivity enhances the integration of the scaffold into bone tissue, making bioprinting a transformative approach for developing treatments for complex bone defects [35,36,42].

Gene therapy integration offers another exciting direction for bone tissue engineering. Gene-activated scaffolds are being developed to deliver therapeutic genes, such as BMP-2, directly to injury sites, promoting localised gene expression and accelerating osteogenesis [74]. By embedding viral or non-viral gene vectors into polymer scaffolds, sustained gene expression can be achieved, reducing the need for large doses of recombinant proteins, which are often expensive and carry risks of side-effects. Non-viral vectors are particularly attractive due to their lower risk profile and compatibility with biodegradable scaffolds [74]. These gene-activated matrices (GAMs) create a bioactive environment conducive to long-term gene expression and enhanced bone regeneration, providing a new method for treating complex bone injuries [74].

In addressing the variability in patient responses to polymer-based scaffolds in clinical settings, several strategies can be employed to enhance outcomes. Personalised medicine allows for tailoring scaffold properties to meet individual patient needs, adjusting factors such as mechanical properties, degradation rates, and bioactivity based on specific patient characteristics like age and health condition. Advanced biomaterials that mimic the natural extracellular matrix (ECM), such as collagen or silk, can improve scaffold integration and reduce adverse responses. Controlled release systems for growth factors within scaffolds can enhance tissue regeneration and modulate the immune response. Extensive in vitro testing using patient-derived cells can predict patient responses and guide customisation of scaffold properties before clinical application. Additionally, scaffolds that modulate the immune response—through coatings with anti-inflammatory agents or the release of immunomodulatory molecules—are critical for reducing adverse reactions. Implementing real-time monitoring systems for scaffold performance and patient response provides valuable feedback, enabling adjustments to treatment plans as needed. By combining these approaches, variability in patient responses can be better managed, significantly improving clinical outcomes and ensuring the success of polymer-based scaffolds in regenerative medicine [75].

Lastly, personalised medicine holds great promise for the future of bone regeneration. Using imaging technologies such as CT scans, highly accurate 3D models of a patient’s bone structure can be generated, allowing for the creation of patient-specific polymer scaffolds customised to the defect’s exact shape and size. This precision ensures the scaffold fits perfectly into the defect site, promoting better mechanical stability and biological integration. The healing process is significantly improved by optimising both the internal architecture and the mechanical properties of these customised scaffolds, particularly in complex fractures or significant bone defects. This approach reduces recovery times and improves clinical outcomes, positioning personalised scaffolds as a critical innovation in advancing bone tissue engineering [76].

Through the continued development of smart polymers, bioprinting, gene therapy, and personalised scaffolds, bone tissue engineering is poised to achieve more effective and personalised treatments for complex bone injuries, ultimately transforming the landscape of regenerative medicine.

## 7. Conclusions

Polymers such as PLGA, PCL, PLA, and chitosan play a crucial role in bone tissue engineering, offering flexible and innovative solutions that can transform regenerative medicine. Their biocompatibility, biodegradability, and adaptability make them essential for promoting bone healing and regeneration. These polymers create scaffolds that mimic the body’s ECM, supporting cell growth, tissue repair, and regeneration. Combining polymers with growth factors, nanoparticles, and other bioactive agents can further enhance these scaffolds, leading to more effective and targeted therapies.

However, despite these advancements, several challenges remain. For instance, some synthetic polymers, like PLA and PLGA, can degrade into acidic byproducts, potentially causing inflammation and toxicity, which requires careful management. Many polymers lack the mechanical strength necessary for load-bearing applications, particularly in significant bone defects. Moreover, not all polymers provide optimal environments for cell attachment and differentiation, requiring further modification to enhance their performance. There are also challenges related to manufacturing complexity and cost, as well as regulatory hurdles such as obtaining FDA approval, which complicate the broader clinical adoption of these materials.

In conclusion, while polymers offer significant potential for bone regeneration and have already demonstrated success in early research, overcoming these challenges is crucial for their widespread use in clinical settings. As ongoing research advances smart polymers, gene therapy, bioprinting, and personalised scaffolds, there is great hope for creating more effective and personalised treatments for complex bone injuries.

## Data Availability

The original contributions presented in the study are included in the article; further inquiries can be directed to the corresponding author.
